# P-281. In-patient mortality trends caused by HIV infection in the United States: A retrospective national database healthcare resource utilization analysis from 2016-2022

**DOI:** 10.1093/ofid/ofaf695.502

**Published:** 2026-01-11

**Authors:** Muhammad Sohaib Asghar, Afsana Ansari Shaik, Maria Duharte, Luis Duharte-Vidaurre

**Affiliations:** AdventHealth Sebring, Sebring, FL; Mayo Clinic, Rochester, Minnesota; South Florida State College, Tampa, Florida; AdventHealth Sebring, Sebring, FL

## Abstract

**Background:**

Despite a total decrease in age-adjusted mortality rate (AAMR) for HIV across all demographic variables, a multitude of factors have accounted for this cumulative effect of reduction in AAMR, including HIV-related hospitalizations, as 59% of these deaths occur in a medical facility. This study’s main objective is to provide insights from the national in-patient database for the recent years regarding the trends of HIV-associated in-hospital mortality and their healthcare resource utilization.

**Figure 1:**
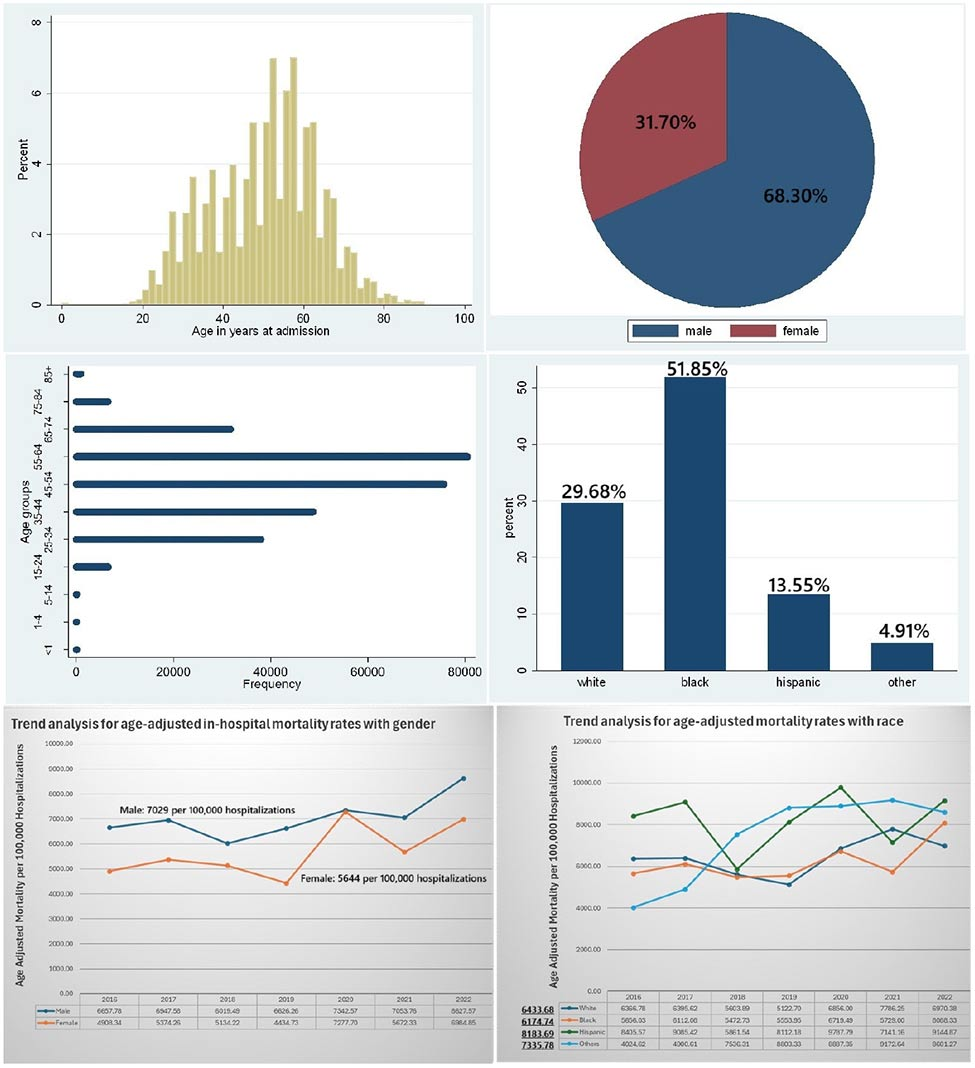
(A). shows age-distribution of HIV-related hospitalizations (n=289,095); (B). gender; (C). age-groups; (D). racial/ethnic groups; (E). Trends in age-adjusted mortality rates among gender; (F). Trends in age-adjusted mortality rates among racial/ethnic groups. (A). Histogram (age). (B). Pie-chart (frequency). (C). Distribution dot-plot (horizontal). (D). Bar graph (vertical). (E). Line graph (two-groups). (F). Line graph (four-groups).

**Figure 2:**
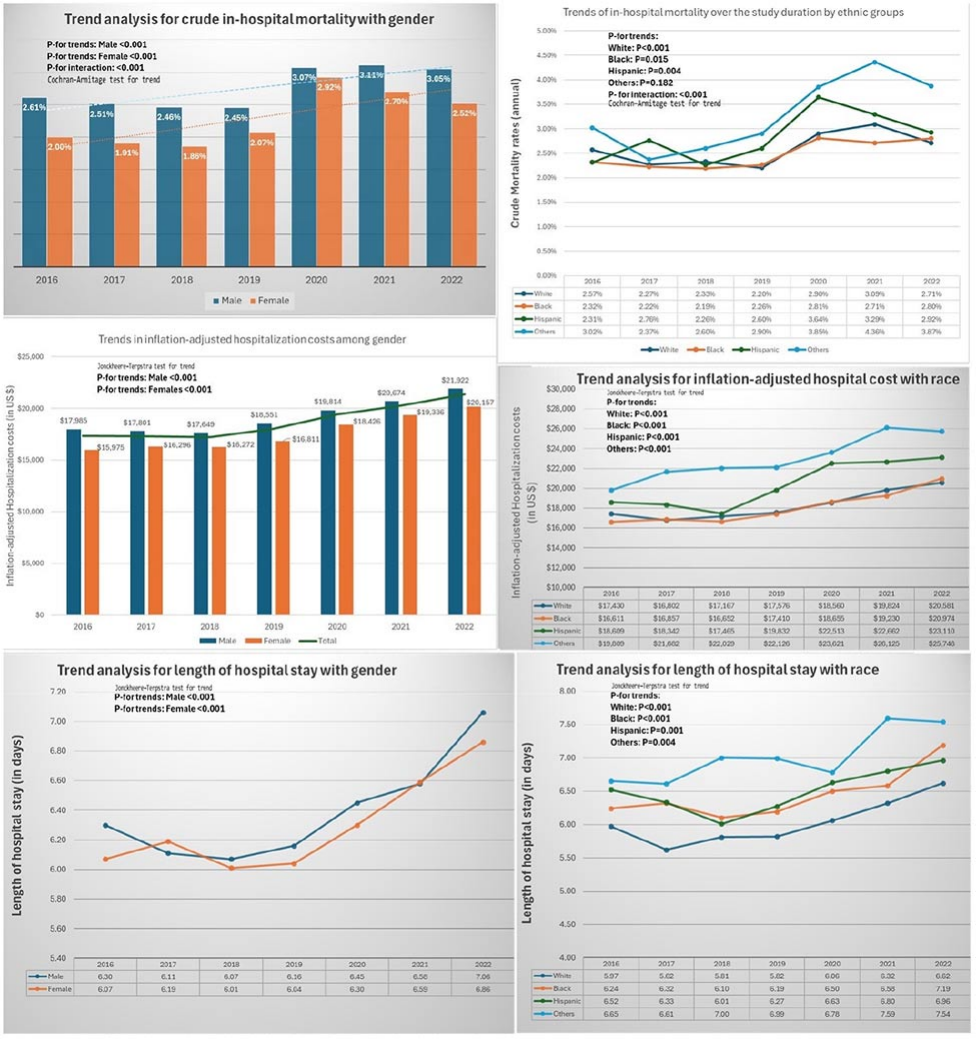
Trend analysis for crude mortality of in-hospital HIV mortality among gender (A), race (B); inflation-adjusted hospitalization cost from 2016-2022 among gender (C), and race (D); and length of hospital stay among gender (E) and race (F). The Cochrane-Armitage test is used for crude mortality rate trend analysis for the years 2016 up to 2022. The Jonckheere-Terpstra test is used for non-parametric distribution of trends in length of stay and hospitalization costs (inflation-adjusted).

**Methods:**

The National Inpatient Sample (NIS) database was searched from 2016 to 2022 to identify patients with HIV (ICD-10 Codes: B20-B24). Hospital discharge weights for national representation were utilized. Baseline social and demographic characteristics along with hospital-level variables were analyzed subsequently using the odds ratio with multivariate regression analysis for mortality adjusted with age. Secondary outcomes included length of hospital stay and inflation-adjusted costs. For cost-based analysis, inflation adjustment was performed according to the inflation index for the Medical Care Consumer Price Index (CPI).

**Figure 3:**
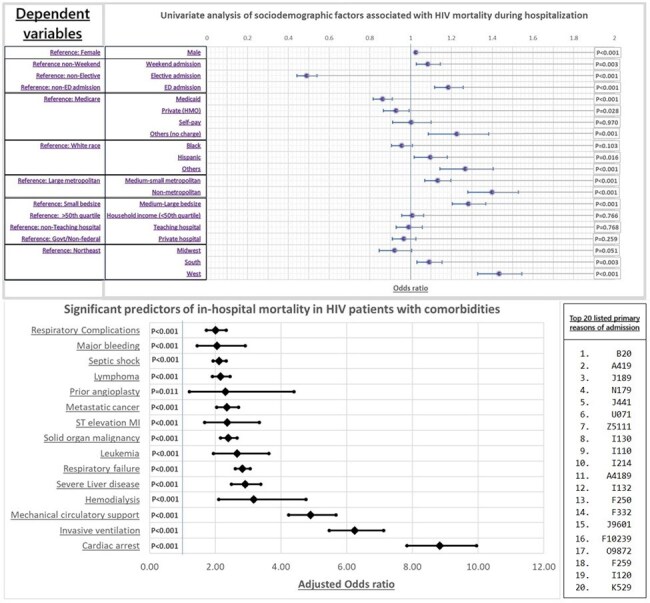
A-Univariate analysis of sociodemographic variables associated with in-hospital mortality with HIV; B-Significant predictors of in-hospital mortality with HIV in patients with comorbidities; C- Top 20 listed primary causes of admission stratified by ICD-10 codes in HIV patients. Top 20 listed primary causes of hospitalization in HIV patients from the year 2016 to 2022: 1. B20 = HIV-related conditions. 2. A41.9 = Sepsis, unspecified organism. 3. J18.9 = Pneumonia, unspecified organism. 4. N17.9 = Acute kidney failure, unspecified. 5. J44.1 = Chronic obstructive pulmonary disease with (acute) exacerbation. 6. U07.1 = COVID-19 infection. 7. Z51.11 = Encounter for antineoplastic chemotherapy. 8. I13.0 = Hypertensive heart and chronic kidney disease (CKD) with heart failure and stage 1 through stage 4 CKD. 9. I10.0 = Hypertensive heart disease with heart failure. 10. I21.4 = Non-ST elevation (NSTEMI) myocardial infarction. 11. A41.89 = Other specified sepsis. 12. I13.2 = Hypertensive heart and chronic kidney disease (CKD) with heart failure and with stage 5 CKD, or end-stage renal disease. 13. F25.0 = Schizoaffective disorder, bipolar type. 14. F33.2 = Major depressive disorder, recurrent severe without psychotic features. 15. J96.01 = Acute respiratory failure with hypoxia. 16. F10.239 Alcohol dependence with withdrawal, unspecified. 17. O98.72 = HIV disease complicating childbirth. 18. F25.9 = Schizoaffective disorder, unspecified. 19. I12.0 = Hypertensive chronic kidney disease (CKD) with stage 5 (CKD) or end-stage renal disease. 20. K52.9 = Noninfective gastroenteritis and colitis, unspecified.

**Table 1:**
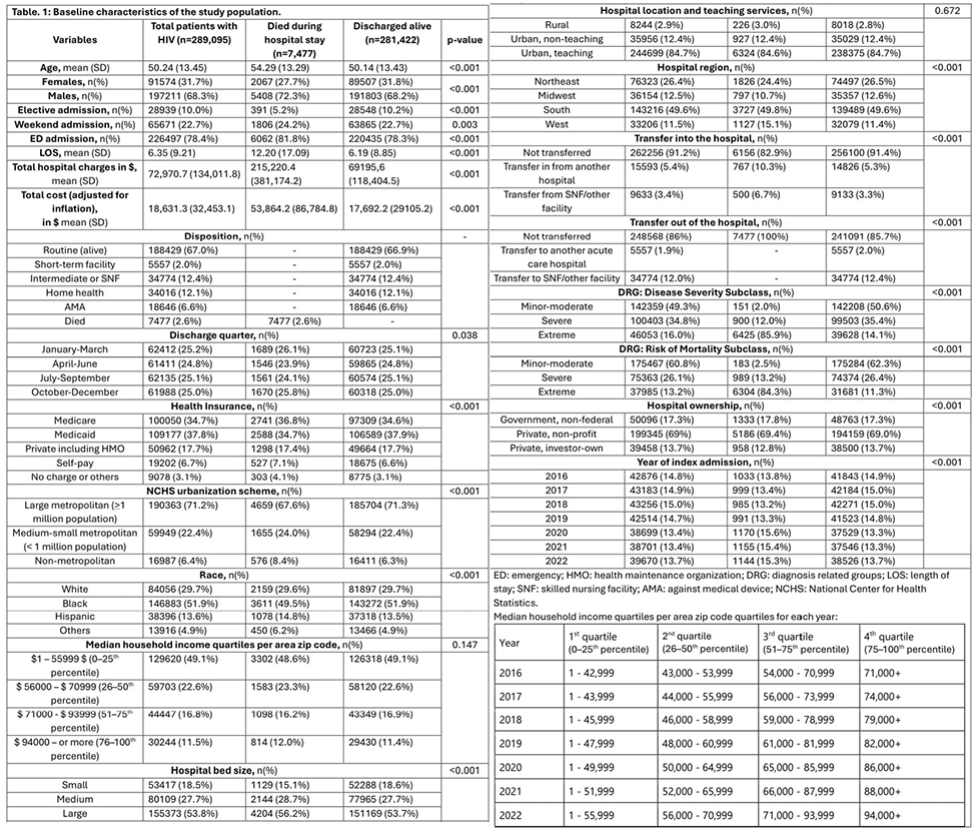
Baseline characteristics of the study population. ED: emergency; HMO: health maintenance organization; DRG: diagnosis related groups; LOS: length of stay; SNF: skilled nursing facility; AMA: against medical device; NCHS: National Center for Health Statistics.

**Results:**

A total of 289,095 HIV-related hospitalizations were included, which represents a weighted size of 1,445,475 hospital discharges. The mean age was 50.24 years, with 68.3% males and 51.85% African Americans. A total of 2.59% in-hospital deaths occurred; AAMR was 7029 per 100,000 hospitalizations in males, which was higher than females (5644 per 100,000). AAMR was found to be highest in the Hispanic population, at 8184 per 100,000. Males had a 3% annual percent change from 2016 to 2022, while trends show an increase in deaths in other races, like Asians/Pacific Islanders/Natives. Most deaths occurred in the West region (OR=1.43), non-metropolitan areas (OR=1.40), and medium-large bed-size hospitals (OR=1.28).

**Conclusion:**

HIV-related in-hospital mortality has posed a significant challenge across various demographic factors. Utilizing mortality data from large databases can serve as a valuable screening tool for targeted public health surveillance and allocation of resources to obtain further development of tailored health programs and interventions aimed particularly at allocation of resources in reducing the impact of HIV-related deaths.

**Disclosures:**

All Authors: No reported disclosures

